# Acid Sensing Ion Channel 2a Is Reduced in the Reduced Uterine Perfusion Pressure Mouse Model and Increases Seizure Susceptibility in Pregnant Mice

**DOI:** 10.3390/cells10051135

**Published:** 2021-05-08

**Authors:** Maria Jones-Muhammad, Qingmei Shao, Loretta Cain-Shields, James P. Shaffery, Junie P. Warrington

**Affiliations:** 1 Program in Neuroscience, University of Mississippi Medical Center, Jackson, MS 39216, USA; mmjones@umc.edu; 2 Department of Neurology, University of Mississippi Medical Center, Jackson, MS 39216, USA; qshao2@umc.edu; 3 Department of Data Sciences, University of Mississippi Medical Center, Jackson, MS 39216, USA; lcain@umc.edu; 4 Department of Psychiatry, University of Mississippi Medical Center, Jackson, MS 39216, USA; jshaffery@umc.edu; 5 Department of Neurobiology and Anatomical Sciences, University of Mississippi Medical Center, Jackson, MS 39216, USA

**Keywords:** eclampsia, placental ischemia, seizure, ASIC2a, pregnancy, RUPP, pentylenetetrazol

## Abstract

Eclampsia is diagnosed in pregnant women who develop novel seizures. Our laboratory showed that the reduced uterine perfusion pressure (RUPP) rat model of preeclampsia displays reduced latency to drug-induced seizures. While acid sensing ion channels (ASIC1a and 3) are important for reducing seizure longevity and severity, the role of ASIC2a in mediating seizure sensitivity in pregnancy has not been investigated. We hypothesized that 1) RUPP reduces hippocampal ASIC2a, and 2) pregnant mice with reduced ASIC2a (ASIC2a+/−) have increased seizure sensitivity. On gestational day 18.5, hippocampi from sham and RUPP C57BL/6 mice were harvested, and ASIC2a was assessed using Western blot. Pregnant wild-type and ASIC2a+/− mice received 40 mg/kg of pentylenetetrazol (i.p.) and were video recorded for 30 min. Behaviors were scored using a modified Racine scale (0–7: 0 = no seizure; 7 = respiratory arrest/death). Seizure severity was classified as mild (score = 1–3) or severe (score = 4–7). RUPP mice had reduced hippocampal and placental ASIC2a protein. ASIC2a+/− mice had reduced latency to seizures, increased seizure duration, increased severe seizure duration, and higher maximum seizure scores. Reduced hippocampal ASIC2a in RUPP mice and increased seizure activity in pregnant ASIC2a+/− mice support the hypothesis that reduced ASIC2a increases seizure sensitivity associated with the RUPP.

## 1. Introduction

Preeclampsia, a hypertensive disorder of pregnancy that affects approximately 5% of pregnancies in the US, can advance to eclampsia, where the mother displays new-onset seizures [1]. Of the 5% of women diagnosed with preeclampsia, about 1.4% are subsequently diagnosed with eclampsia [1], which is responsible for roughly 10% of maternal deaths worldwide. The number of (pre)eclampsia cases is growing [2,3,4]. Women who survive preeclampsia/eclampsia complications are at a higher risk for cognitive decline later in life [5], and their children are at a higher risk of developing high blood pressure and other cardiovascular diseases, in addition to neurodevelopmental disorders [6,7]. In some cases, eclampsia can be avoided if the fetus and placenta are delivered early [8]. However, there are cases of women experiencing eclampsia during the postpartum period [9] and early delivery increases the number of premature births, which is also associated with adverse outcomes. Due to the detrimental impacts of eclampsia on the mother and fetus, it is imperative that the underlying mechanisms are identified.

Why some women, with otherwise healthy pregnancies, go on to have novel seizures is still unknown. In the clinic, women diagnosed with preeclampsia with severe symptoms are given magnesium sulfate prophylactically to prevent the progression to eclampsia. While magnesium sulfate is effective in preventing seizures, it cannot be used for chronic treatment, and the mechanism of magnesium sulfate’s anti-seizure activity is not fully understood. Moreover, treatment with magnesium sulfate does not work in all patients [10]. In order to develop better treatments for eclampsia, a greater understanding of the mechanisms underlying the transition from preeclampsia to eclampsia is needed.

In preclinical experimental studies, seizures are induced in animal models of preeclampsia to produce eclampsia models. Pentylenetetrazol (PTZ), a GABA receptor antagonist previously demonstrated to produce temporal lobe epilepsy [11], is commonly used in preclinical eclampsia models [12,13,14]. Previous research from our group used PTZ to induce seizures in RUPP rats and showed reduced latency to seizure activity. This implies increased seizure susceptibility in RUPP rats compared to normal pregnant controls [13]. The mechanisms by which the RUPP procedure increases seizure susceptibility have not been fully elucidated. 

One potential mechanism could be reduced acid sensing ion channel (ASIC) expression and activity. ASICs, proton-gated ion channels found in neural and peripheral tissue, are activated by reductions in the extracellular pH, a physiological response to ischemia and epilepsy. ASIC1a and 3 have important roles in mediating seizure activity [15]. The role of ASIC2a in seizure activity is not fully known, given that it is activated at a lower threshold of acidity (pH 4.0–4.9) [15]. Interestingly, Wu et al. reported that following induced seizures in male rats, ASIC2a expression was upregulated [16]. Moreover, overexpression of ASIC2a in the hippocampus of male rats increased seizure susceptibility [17]. Whether ASIC2a expression is altered in the RUPP model and whether the knockdown of ASIC2a in pregnant mice induces increased seizure severity and duration are not known. 

Thus, the objectives of the current study were to: (1) determine whether RUPP surgery in mice induces decreased ASIC2a protein expression in the hippocampus and placenta; (2) determine whether ASIC2a heterozygous knockout mice have increased seizure severity and longevity during late pregnancy.

## 2. Materials and Methods

### 2.1. Animals

In this study, 10- to 12-week-old timed-pregnant C57BL/6 female mice (*n* = 11) from Charles River Laboratories were used for establishing the RUPP model in mice and for analysis of placental and hippocampal ASIC2a expression. Heterozygous B6.129-Asic2tm1Wsh/J mice were obtained from Jackson Laboratory (JAX Mice stock number: 013126) [18] and bred in the Lab Animal Facilities at the University of Mississippi Medical Center (UMMC) to generate genotypes for the experiments. Mice were fed a standard rodent diet (Teklad 22/5 Rodent Diet 8640) and water ad libitum until breeding, when they were switched to a breeders’ diet (Teklad global 19% protein extruded rodent diet 2019) from mating until euthanasia at gestational day (GD) 18.5. Mice were housed at 2–5 per cage at a constant temperature of 74 ℉ and a 12 h light and 12 h dark cycle. All procedures were approved by the UMMC Institutional Animal Care and Use Committee (Protocol: 1434, 1434A, and 1509) in accordance with the 8th edition of the National Research Council Guide for Care and Use of Laboratory animals 

### 2.2. Breeding and Establishment of Timed Pregnancy for ASIC2a Mice

One to 3 female mice were paired with 1 male of the same age and genotype in the evening. The following day, male mice were returned to their home cages. As mice were paired for only 1 night, the exact date of pregnancy is known. The day of separation was considered as GD 0.5. Mice were monitored for changes in abdominal size, and pregnancy was confirmed on GD 13.5. Mice that did not become pregnant were subjected to one additional round of mating.

### 2.3. Mouse RUPP Model

On gestational day 13.5, mice were anesthetized using 3% isoflurane (2% maintenance). A modified RUPP procedure, which has been demonstrated elsewhere [19], was used to induce utero-placental ischemia. An abdominal incision was made after cleaning with bromide and 70% ethanol, and the uterus containing the placental–fetal unit was exteriorized. The uterine arteries between the ovaries and first pup were ligated on both uterine horns using braided silk sutures (5.0) in RUPP mice (*n* = 7). Uterine horns were exposed without occlusion in sham mice (*n* = 6). Following surgery, all mice were given an analgesic (5.0 mg/kg Carprofen, s.c.) on the day of surgery and Rimadyl tablets daily for three days following the surgery. Our a priori exclusion criteria included excluding dams with only 1 pup present at GD13.5, mice that were not pregnant, and mice with 100% fetal resorptions on GD18.5 (*n* = 1 from the RUPP group). Additionally, mice with pups on only 1 horn were assigned to the sham group. Mice were not excluded from analysis for not having a preeclampsia phenotype. 

### 2.4. Euthanasia and Tissue Collection

At gestation day (GD) 18.5, mice were weighed and anesthetized using 3% isoflurane, and blood was collected via cardiac puncture. Organs were removed and weighed. The number of live and resorbed pups were counted, and pups and placentas were weighed. Blood samples were collected from the dams and pups and used for assessing hematocrit, which has been used in previous studies to assess whether an animal is in a hypoxic state [20]. Brains were removed and dissected to isolate the hippocampus, which was flash frozen in liquid nitrogen and stored at −80 °C until further processing. The mean pup weight, placenta weight, and pup hematocrit were calculated per dam. 

### 2.5. ELISA Analysis

Blood samples were centrifuged at 2000 rpm for 20 min, and serum was collected and stored at −20 °C until processing. Quantikine® ELISA kits from R&D systems and Biotechne were used. Each kit was specific for vascular endothelial growth factor (VEGF, Catalog no. MMV00), VEGFR1 or Flt-1 (Catalog no. MVR100), and placental growth factor (PlGF2, Catalog no. MP200). According to the manufacturer’s information, the mouse VEGFR1/Flt-1 ELISA kit measures natural and recombinant mouse sVEGFR1 (sFlt-1). Maternal serum was diluted with the dilution buffer included in the kit as follows: 1:20 for Flt-1, 1:4 for VEGF, and 1:4 for PlGF. Standards and samples were assayed in duplicate in 96-well plates coated with the appropriate polyclonal antibody following the manufacturer’s instructions. 

### 2.6. Western Blot Analysis

Placental and hippocampal tissues from sham and RUPP mice were homogenized using ChemCruz RIPA lysis buffer in a mini bead homogenizer at 4000 rpm. The supernatant for each sample was collected. A bicinchoninic acid (BCA) protein assay was used to determine the amount of protein in each sample. Samples were prepared for Western blot analysis using 1x PBS, 4X sample buffer which contained 2 beta-mercaptoethanol. Samples were electrophoresed through Criterion TGX 4–20% Stain-free gel at 120 V for 90 min. When completed, the gel was activated using the ChemiDoc MP Imaging System for 45 s. The proteins were transferred to nitrocellulose membranes using the Trans blot turbo transfer kit. Following 30 min of blocking in Odyssey blocking buffer, the primary antibodies for the proteins of interest (ASIC2a antibody, 1:1000, Thermo Fisher Scientific, catalog# OSR00097W) and alpha-tubulin antibody (1:15,000, Abcam, catalog# ab89984) were added and incubated overnight. After washing, the secondary antibodies, donkey anti rabbit (1:15,000, Li-Cor, Catalog# 131591), and donkey anti chicken (1:15,000, Li-Cor, Catalog# 925-68028) were added. Membranes were imaged using the Chemidoc MP system. Protein expression was normalized to alpha tubulin expression. Blots were analyzed using the Bio-Rad Chemidoc Image Lab software. 

### 2.7. Measurement of Hypoxia-Inducible Factor in Placental Samples

Placental homogenates were assayed in duplicate on a HIF1α ELISA kit (Human/Mouse total HIF1A ELISA, Cat #: DYC1935-2, R&D Systems) following the manufacturer’s directions. Samples were run undiluted. Measured concentrations were normalized to total protein in each sample to obtain pg/mg protein.

### 2.8. Seizure Induction Procedure

On GD 18.5, pregnant ASIC2a+/+ (*n* = 7) and ASIC2a+/− (*n* = 14) mice were administered 40 mg/kg of pentylenetetrazol (PTZ) via intraperitoneal (i.p.) injection. After injection, animals were video monitored for 30 min. The observer was blind to the genotype. Behavior was scored using a revised version of the Racine seizure scoring scale [21] from 0 to 7, with 0 indicating no seizure or normal behavior and 7 indicating respiratory arrest. Seizure activity was analyzed using the Observer XT software (Noldus Information Technology, Leesburg, VA, USA). We measured latency to the first seizure behavior, the total duration of seizure activity, the total duration of and latency to severe seizures (score 4–7), and the highest seizure score of each animal throughout the behavior monitoring. Immediately after observation, mice were euthanized and organs harvested under isoflurane anesthesia.

### 2.9. ASIC2a Genotyping

Tail snips were collected and used to confirm ASIC2a genotype. The KAPA Mouse Genotyping Kit from KAPABIOSYSTEMS was used for DNA extraction and PCR genotyping. The protocol for using KAPPA Expression Extract required a 50 µL solution comprising 44 µL of di-deionized water, 5 µL of 10X KAPA Expression Extract Buffer, and 1 U/µL of KAPA E. E. Enzyme. The tail snip was added to the solution, and the tissue was lysed at 75 °C for 10 min. The enzyme was inactivated at 95 °C for 5 min. After lysis, the lysate was centrifuged for 5 min, and the supernatant was stored for genotyping. The following sequences were used (5′–3′): Reaction A: AGT CCT GCA CGG TGG GAG CTT CTA; GAA GAG GAA GGG AGC CAT GAT GAG; Reaction B: ATG GTT TCG GAG TGG TTT GGC ATT GTG and TGG ATG TGG AAT GTG TGC GA. (Heterozygous band: 365 and 450 bp; wild type: 365 bp.) Gels for running samples were 2% gels comprising 35 mL of di-deionized water, 0.7 g of agarose gel, and 3.5 µL of GelRed. An amount of 6 µL of 100 bp ruler was loaded, and 5 µL of sample was loaded before running the gel at 140 V for 45 min. 

### 2.10. Data Analysis

Datasets were tested for normality using the Shapiro–Wilk test. We used Mann–Whitney U tests for non-normal data distributions and an unpaired *t*-test for normally distributed datasets. Data are presented as the mean ± SD. Differences were considered statistically significant at the *p* < 0.05 level. One mouse from the RUPP group was excluded from analysis due to having 100% resorptions at GD18.5 as a result of the surgery. Statistical analyses were performed, and graphs were generated using GraphPad Prism software (version 8.4.3).

## 3. Results

### 3.1. The Mouse RUPP Model Has Some Similar Clinical Characteristics to the Preeclampsia Patient and RUPP Rat

The maternal and fetal characteristics of the RUPP model at GD 18.5 are summarized in Table 1. There was no significant difference in maternal body weight or maternal hematocrit between groups at GD18.5. Additionally, at GD13.5, before the surgeries, there was no difference in body weight between the dams (28.0 ± 1.6 in dams designated to sham group vs. 29.5 ± 0.9 in the dams subjected to the RUPP; *p* = 0.218). In terms of fetal outcomes, there was a reduction in the number of live pups, and no difference in fetal resorptions, pup weight, or placental weight. Fetal hematocrit was increased in the RUPP group, indicating fetal hypoxia. 

**Figure 1:**
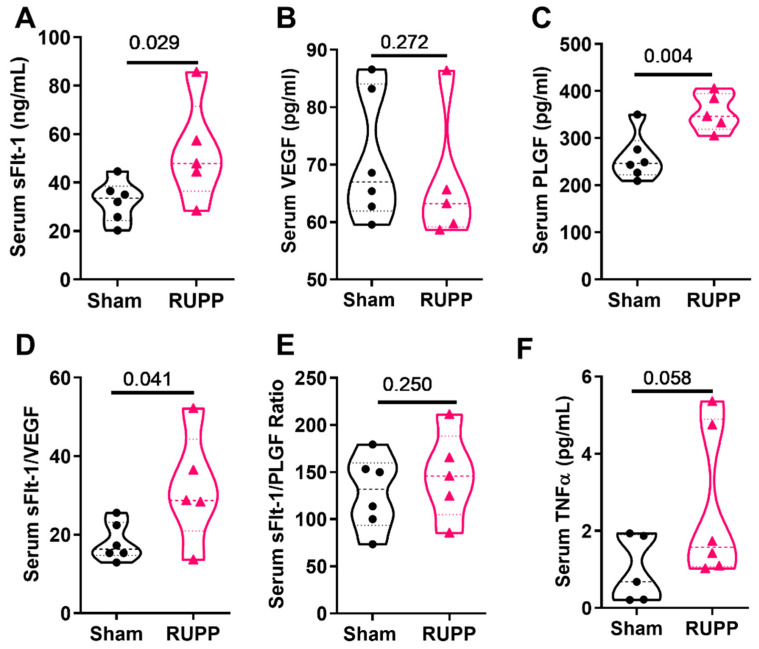
RUPP in the mouse induces some similar changes in angiogenic factors to the rat and preeclampsia patient. RUPP-induced (**A**) increased sFlt-1, (**B**) no change in VEGF, (**C**) increased PlGF, (**D**) increased sFlt-1/VEGF ratio, and (**E**) no change in sFlt-1/PlGF ratio in serum samples compared to sham controls. (**F**) Trend for increased TNFα levels in RUPP mice. Sham *n* = 6 mice and RUPP *n* = 5. Data analyzed using unpaired *t*-test. Points indicate values for individual dams. SFlt—soluble Fms-like tyrosine kinase 1; VEGF—vascular endothelial growth factor; PLGF—placental growth factor.

To further characterize the mouse RUPP model, we measured maternal circulating factors using serum samples. The serum concentration of sFlt1 was significantly increased in RUPP mice when compared to sham mice (52.73 ± 9.5 vs. 32.39 ± 3.5 pg/mL; *p* = 0.0289; Figure 1A), while the VEGF concentration was not different between groups (71.04 ± 11.22 vs. 66.7 ± 11.35 pg/mL; *p* = 0.272; Figure 1B). RUPP mice had a higher serum concentration of PlGF (354.8 ± 18.02 vs. 259.2 ± 20.36 pg/mL; *p* = 0.0037; Figure 1C), a higher sFlt-1/VEGF ratio (31.87 ± 14.05 vs. 18.14 ± 4.840; *p* = 0.025; Figure 1D), and no difference in the sFlt-1/PlGF ratio (146.4 ± 46.77 vs. 128.3 ± 39.24; *p* = 0.250; Figure 1E). In response to RUPP, mice showed an increasing trend for circulating TNFα levels (2.56 ± 1.95 in RUPP vs. 0.98 ± 0.86 pg/mL in sham; *p* = 0.058; Figure 1F). Placental HIF1α was not different between the groups (3.90 ± 0.71 pg/mg protein in shams vs. 3.91 ± 0.60 pg/mg protein in RUPP; *p* = 0.497). Together, these data demonstrate that the mouse RUPP model displays few similar features of the rat RUPP model and clinical preeclampsia, with elevated sFlt-1 being the primary shared clinical characteristic. 

### 3.2. The RUPP Model in the Mouse Induced Reduced ASIC2a Protein Expression in the Placenta and Hippocampus

**Figure 2:**
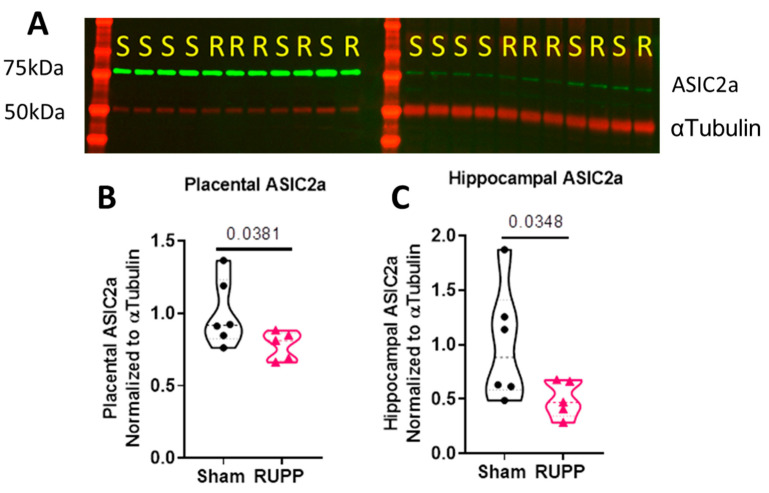
RUPP surgery reduces hippocampal and placental expression of ASIC2a. (**A**) Representative Western blot image from placental and hippocampal samples. Mice that underwent the RUPP procedure displayed reduced (**B**) placental and (**C**) hippocampal ASIC2a expression. Data were normalized to alpha tubulin followed by normalization to the sham levels. SHAM *n* = 6; RUPP *n* = 5. Data were analyzed using unpaired *t*-test (**B**) or Welch’s *t*-test (**C**). Points indicate values for individual dams.

Western blot analysis was used to determine if RUPP induces changes in the expression of ASIC2a in the hippocampus and placenta. In the hippocampus, there was a significant reduction in the expression of ASIC2a in RUPP mice compared to the sham control (0.49 ± 0.08 vs. 1.0 ± 0.22; *p* = 0.037; Figure 2A). In the placenta, RUPP mice had a reduced expression of ASIC2a compared to sham mice (0.78 ± 0.04 vs. 1.0 ± 0.09; *p* = 0.038; Figure 2B). Full images of the Western blots can be found in the Supplementary Materials Figures S1–S3. These data show that the RUPP procedure induced a reduction in ASIC2a expression in the hippocampus and placenta.

### 3.3. General Characteristics of Pregnant ASIC2a Wild-Type and Heterozygous Knockout Mice at GD18.5

Pregnant ASIC2a +/+ and +/− mice were used for assessing seizure susceptibility. A representative gel for each genotype can be found in Supplementary Materials Figure S4. Pregnancy characteristics for each genotype are shown in Table 2. There was no difference in body weight between +/+ and +/− dams, the number of live pups, fetal resorptions, pup weight, or placental weight at GD 18.5. 

### 3.4. Knockdown of ASIC2a Results in Increased Seizure Severity and Longevity during Late Pregnancy

**Figure 3:**
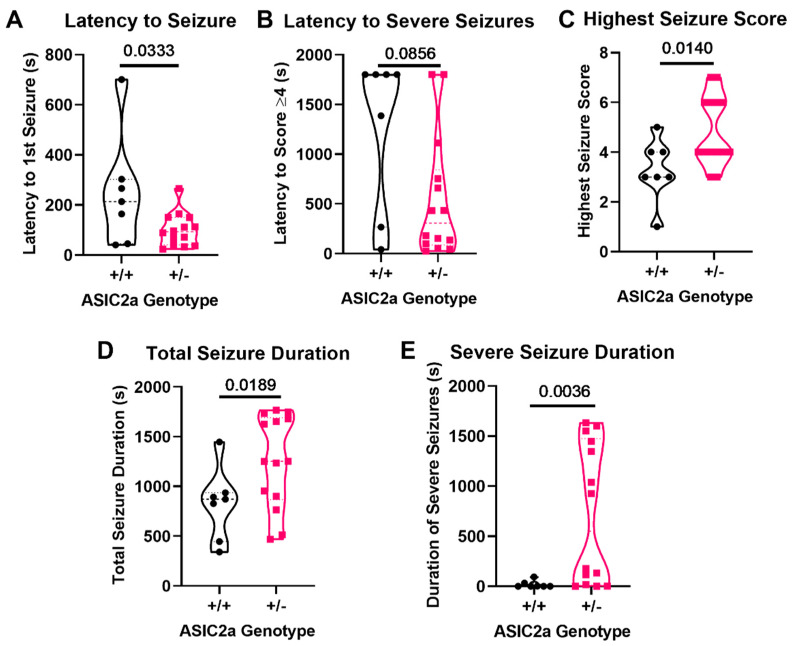
ASIC2a knockdown results in increased seizure severity and longevity in pregnancy. Pregnant ASIC2a+/− mice show (**A**) significantly reduced latency to the first seizure activity, (**B**) no difference in reduced latency to severe seizure activity, (**C**) higher seizure scores, (**D**) increased total duration of seizures, and (**E**) increased duration of severe seizures compared to pregnant ASIC2a+/+ mice. *n* = 7 (+/+) or *n* = 14 (+/−) mice. Data analyzed using Mann–Whitney U test. Points indicate values for individual dams.

The Racine scale was used to analyze seizure behavior in ASIC2a mice. Pregnant ASIC2a +/− mice have significantly reduced latency to the first detected seizure behavior compared to pregnant +/+ mice (101.4 ± 17.61s vs. 247.7 ± 84.57s *p* = 0.033; Figure 3A). There was no differences in the latency to severe seizures (*p* > 0.05; Figure 3B); however, +/− mice had a higher overall seizure score compared to +/+ mice (4.9 ± 0.4 vs. 3.3 ± 0.5 *p* = 0.014; Figure 3C). ASIC2a+/− mice had an overall longer duration of seizure than +/+ mice (1253 ± 124.7s vs. 822.6 ± 136.6s *p* = 0.019; Figure 3D) as well as longer severe seizures (713.1 ± 187.7s vs. 18.29 ± 13.38s *p* = 0.0036; Figure 3E). These results indicate that reduced ASIC2a expression increases seizure susceptibility and severity during pregnancy.

## 4. Discussion

The mechanisms contributing to increased seizure sensitivity following RUPP are not fully known. The current study investigated whether inducing RUPP in mice would lead to a decrease in ASIC2a expression and whether the knockdown of ASIC2a would lead to increases in seizure susceptibility during normal pregnancy. We first measured the expression of ASIC2a in placental and hippocampal tissues from sham and RUPP mice and then determined whether reduced ASIC2a expression increased seizure severity and longevity in pregnant mice. The results indicate that RUPP mice have a reduction in ASIC2a protein expression in the hippocampus and the placenta at gestational day 18.5 of pregnancy. When seizures were induced in pregnant ASIC2a+/+ and +/− mice, ASIC2a+/− mice displayed seizure behavior suggesting increased seizure susceptibility and severity. These data support the hypothesis that the reduced hippocampal expression of ASIC2a in RUPP mice is a contributing factor to RUPP-induced increases in seizure susceptibility, although this hypothesis remains to be directly tested.

### 4.1. The Mouse RUPP Model

The current study used a version of the RUPP described by Fushima et al. where the authors reported several characteristics similar to the preeclampsia patient and the rat RUPP model [19]. While the majority of studies utilizing RUPP surgery are performed in rats [22,23,24,25], the use of a mouse RUPP model allows investigators to directly test the involvement of specific pathways by using genetic mouse models. Hallmarks of the rat RUPP model mimic features of clinical preeclampsia and include increased blood pressure; increased fetal demise; and an imbalance of maternal angiogenic factors PlGF, VEGF, and sFlt1 [26,27,28,29]. Here, we demonstrated that pregnant mice subjected to the RUPP procedure, as presented by Fushima et al. [19], display fewer similar features. We demonstrate fetal hypoxia (increased fetal hematocrit), reduced litter size at GD18.5, elevated sFlt-1, and a trend for increased TNFα in maternal serum. We do not know why we were not able to replicate all the findings reported in the Fushima study but think that the difference in the length of exposure to the RUPP may have contributed. In the study by Fushima and colleagues, the length of ischemia was 4 days (GD14–18), while our model was exposed for 5 days (GD13.5–18.5). Moreover, in our model, while hematocrit was increased in the fetuses, we did not find evidence of placental hypoxia, as placental HIF-1α was not different between the groups. We did not measure proteinuria or blood pressure from conscious mice and did not find evidence of intrauterine growth restriction in this study. It is possible that occluding only the uterine arteries near the ovaries resulted in compensatory flow from the abdominal aorta, supplying the inferior uterine horn. This compensatory flow could, therefore, contribute to preserving fetal weights. Indeed, in preliminary studies and published work, when the abdominal aorta is partially occluded, intrauterine growth restriction results [19,30]. We chose not to occlude the abdominal aorta in this study due to the complication of hind limb paralysis that occurred in a proportion of the mice used in pilot studies (data not shown). 

The RUPP model is used in many studies investigating preeclampsia due to its ability to re-create symptoms of clinical preeclampsia, such as reduced concentrations of VEGF and PlGF in urine and maternal serum [31,32,33,34]. Although the modified RUPP model displayed similar symptoms to that of the rat RUPP model, such as an increase in serum sFlt-1 concentration, we found a significant increase in the concentration of PlGF, no change in the concentration of VEGF, and an increase in the sFlt1/VEGF ratio in maternal serum, whereas other models display a significant reduction in PlGF and VEGF [24,27]. It should be noted that the studies investigating changes in PlGF and VEGF were performed in rats and analyzed free or unbound VEGF and PlGF levels, whereas the ELISA kit in the current study measured total VEGF and PlGF. Importantly, when the ratio of sFLt-1/VEGF was calculated, we found a significant increase in the RUPP, suggesting that the level of unbound and bioavailable VEGF is reduced in response to the RUPP. The use of an ELISA kit that quantifies the unbound proangiogenic factors will aid in better characterizing the RUPP model. 

### 4.2. Reduced ASIC2a Expression

ASICs are mechano- and chemoreceptors that have a significant role in neuroprotection [35,36]. The ability of ASICs to sense reductions in the extracellular pH, which occurs in adverse circumstances, such as seizures or hypertension [16,35,37], suggests that they may have a role in seizure susceptibility induced by RUPP. Several studies have shown that manipulating various ASIC isoforms via genetic and pharmacological approaches (ASIC1a reduction and ASIC3 elevation) increases seizure severity [38,39,40]. Studies investigating the role of ASIC2a show that ASIC2a overexpression increases seizure severity [16,17]. In spite of this phenomenon, there are no studies that have investigated the impact of reduced ASIC2a on seizure susceptibility in females. Additionally, no studies have investigated changes in ASIC2a expression in normal pregnancy or following the RUPP procedure. Due to this gap in knowledge, we assessed whether RUPP contributed to changes in ASIC2a expression.

It is currently not known what factors contribute to seizures in pregnancy and preeclampsia. This gap in knowledge is critical, given the life-threatening and long-term health risks of the mother following preeclampsia [41] and the dangers a seizure during pregnancy poses on the fetus [42]. Women with severe preeclampsia are given magnesium sulfate to prevent seizures; however, this method is not preventative in all cases of preeclampsia. Additionally, finding the optimal point at which treatments can prevent seizure as well as prevent the need to induce early delivery is imperative. Previous work from our laboratory demonstrated increased seizure susceptibility in a rat RUPP model [13]; however, the mechanism by which this susceptibility occurs had not been investigated. Here, we demonstrated, for the first time, that the pH-activated channel ASIC2a is significantly reduced in the placenta and hippocampus of the mouse RUPP model. Investigating seizure susceptibility in the mouse RUPP model is required and is being investigated in the lab. Additionally, whether manipulating ASIC2a expression in RUPP mice modulates seizure activity has not been investigated and is the next logical research question to answer. 

Our finding of reduced ASIC2a expression in the placenta is interesting. Currently, there are no studies investigating the role of ASIC2a in the placenta. Nevertheless, work by Ali and colleagues showed that reductions in the extracellular pH led to marked vasodilation in placental arteries isolated from healthy pregnant women and that the response to reduced pH was markedly attenuated in vessels isolated from preeclampsia patients [43]. These findings, along with the finding of reduced ASIC2a in the RUPP placenta of our current study, suggest that pH-sensitive signaling is impaired in placentas from preeclampsia patients, and this may be due to a reduction in the expression of ASICs, such as ASIC2a. 

### 4.3. Seizures in Pregnant ASIC2a Mice 

The current findings demonstrate that pregnant mice with reduced ASIC2a have increased seizure susceptibility. Previous findings have suggested that the overexpression of ASIC2a increases seizure activity as well; however, the study used male rats in which ASIC2a was overexpressed in the hippocampus, whereas the current study used pregnant female mice with a global reduction in ASIC2a [16,17]. Whether there are sex- and species-dependent differences in the effect of ASIC2a manipulation on seizure susceptibility requires further investigation. It is also possible that there is a specific level of ASIC2a required for normal neuronal activity and that increasing or decreasing beyond that level would result in increased seizure susceptibility. The possibility of pregnancy having a significant impact on seizure susceptibility cannot be ruled out. Indeed, previous work by Johnson et al. found that normal pregnancy contributes to increased seizure susceptibility when compared to the non-pregnant control [14]. Although the previously mentioned study used rats, determining the contribution of pregnancy to seizure susceptibility in mice requires further investigation. 

Another study analyzed the expression of ASIC2a in human hippocampal samples obtained from epilepsy patients and found elevated ASIC2a expression. Of note, only patients with a history of epilepsy were analyzed [17]; therefore, it is not known if these ASIC2a levels were gradually elevated as the epilepsy disorder progressed or whether ASIC2a levels were already different before the development of the seizure disorder. As the patients had a history of epilepsy, these human data may correspond to women who have epilepsy before becoming pregnant rather than de novo seizures during gestation or in the postpartum period [17]. We did not assess whether ASIC2a expression increases following seizures similarly to what is observed in epilepsy patients. This question will be investigated in future studies. 

### 4.4. Limitations

The limitations of our study need to be considered. We did not measure blood pressure in our mice and cannot confidently say whether our findings were influenced by blood pressure changes in the RUPP. Moreover, we did not determine whether RUPP mice have evidence of proteinuria or kidney injury or even some of the cerebrovascular abnormalities reported in RUPP rats. Further characterization of the mouse RUPP model is required. In addition, to continue characterizing our RUPP mouse model, future work will investigate whether seizure induction using PTZ in RUPP mice will replicate the findings in the rat RUPP model. We will also need to investigate whether manipulating the expression of ASIC2a and inducing RUPP in these mice will lead to changes in seizure severity and longevity.

Another important consideration is that other isoforms of ASICs may also be affected by the RUPP procedure. ASIC2a forms complexes with other ASIC isoforms, such as ASIC2b and ASIC1a, and epithelial sodium channel (beta ENaC) proteins [15,44,45]. Ongoing studies are investigating whether RUPP leads to changes in the expression of these proteins. Another caveat to consider is that we used only one method to induce seizures and to analyze seizure behavior. Future studies will incorporate the use other pro-convulsive drugs and EEG recordings for further confirmation of seizure activity, similar to other studies [46].

## 5. Conclusions

In conclusion, our findings are the first, to our knowledge, to demonstrate that RUPP mice have a reduced expression of hippocampal and placental ASIC2a. This study is also the first to show that a reduction in ASIC2a expression leads to more severe seizure activity and increased duration of seizures during pregnancy. Thus, ASIC2a may be a therapeutic target to prevent the occurrence or to reduce the severity of seizures in pregnancy. Further investigation is required to determine if RUPP and/or ASIC2a reduction contributes to changes in the expression of excitatory and inhibitory neurotransmitter receptors or other ASIC isoforms in the context of epilepsy.

## Figures and Tables

**Table 1:** General characteristics and pregnancy outcomes in RUPP mice.

Characteristic	Sham (*n* = 6)	RUPP (*n* = 6)	*p*-Value
Maternal			
Body weight (g)	39.2 ± 1.4	37.8 ± 1.9	0.289
Hematocrit (%)	35 ± 2	35 ± 1	‡ 0.446
Fetal Outcomes			
No. of live pups	8 ± 1	5 ± 1	0.0499 †
% resorptions	0 ± 0	21 ± 15	0.111
Pup weight (g)	1.13 ± 0.03	1.21 ± 0.06	§ 0.294
Placenta weight (g)	0.09 ± 0.00	0.10 ± 0.00	0.173
Pup Hematocrit (%)	30 ± 2	35 ± 1	0.037 †

† *p* = 0.0499 vs. sham; ‡ data analyzed using Welch’s test; § data analyzed using Mann–Whitney U test.

**Table 2:** General characteristics and pregnancy outcomes in ASIC2a mice at GD18.5.

Characteristic	ASIC2a+/+ (*n* = 7)	ASIC2a+/− (*n* = 14)	*p*-Value
Maternal			
Body weight (g)	35.5 ± 2.2	35.4±3.0	0.406
Fetal Outcomes			
No. of live pups	8 ± 1	7 ± 2	0.358
% resorptions	11 ± 13	5 ± 6	0.071
Pup weight (g)	0.87 ± 0.02	0.85 ± 0.01	0.096
Placenta weight (g)	0.10 ± 0.01	0.11 ± 0.01	0.066

Data analyzed using Mann–Whitney *U* test.

## Data Availability

All data are available from the corresponding author upon reasonable request.

## Supplementary Materials

The following are available online at https://www.mdpi.com/article/10.3390/cells10051135/s1, Figure S1: Full Western blot for ASIC2a (green) and alpha tubulin (red) staining. Membrane was probed for both ASIC2a and alpha tubulin. Placental samples are shown on the left, while hippocampal samples are shown on the right. The ASIC2a bands were observed at ~76 kDa, and alpha tubulin bands were visible at 50 kDa. Figure S2: Full Western blot (inverted greyscale image) for ASIC2a staining. On the left, placental samples were loaded. On the right, hippocampal samples are shown. The ASIC2a bands were observed at ~76 kDa. Figure S3: Full Western blot (inverted greyscale image) for alpha tubulin staining. On the left, placental samples were loaded. On the right, hippocampal samples are shown. The antibody recognized a band at 50 kDa. Extra bands were visible in the hippocampal samples. Figure S4: Representative bands from ASIC2a genotyping. ASIC2a+/+ mice have only the WT band (365bp), while ASIC2a+/− mice have the WT band plus the mutant band (450 bp).
