# Supplementary material for: Acid Sensing Ion Channel 2a Is Reduced in the Reduced Uterine Perfusion Pressure Mouse Model and Increases Seizure Susceptibility in Pregnant Mice
Source: Cells. 2021 May 8;10(5):1135. doi: 10.3390/cells10051135 (PMC8151496; doi:10.3390/cells10051135)
Supplement: Supplementary file 1 [file cells-10-01135-s001.zip › cells-1175142-supplementary.pdf]

cells

MDPI

Supplementary Materials:

Reduced Acid Sensing Ion Channel 2a increases Seizure Susceptibility in Pregnant Mice: Role in Utero-placental Ischemia

Maria Jones-Muhammad [1], Qingmei Shao [2], Loretta Cain-Shields [3], James P. Shaffery [4], Junie P. Warrington[2,5,*]

[1] Program in Neuroscience; University of Mississippi Medical Center, Jackson, MS 39216
[2] Department of Neurology; University of Mississippi Medical Center, Jackson, MS 39216
[3] Department of Data Sciences; University of Mississippi Medical Center, Jackson, MS 39216
[4] Department of Psychiatry; University of Mississippi Medical Center, Jackson, MS 39216
[5] Department of Neurobiology and Anatomical Sciences; University of Mississippi Medical Center, Jackson, MS 39216
* Correspondence: jpwarrington@umc.edu; Tel.: 1-601-815-8969

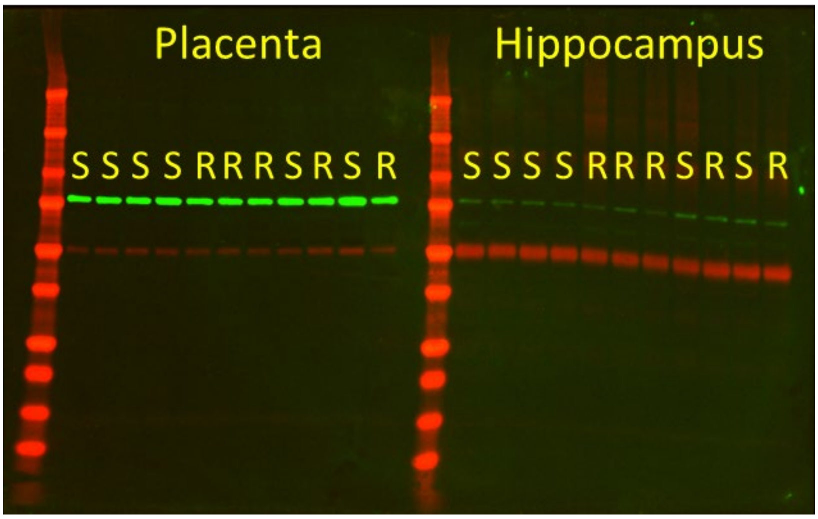

Figure S1: Full Western blot for ASIC2a (green) and alpha tubulin (red) staining. Membrane was probed for both ASIC2a and alpha tubulin. Placental samples are shown on the left while hippocampal samples are shown on the right. The ASIC2a bands were observed at ~76kDa and alpha tubulin bands were visible at 50kDa. S- Sham, R-RUPP

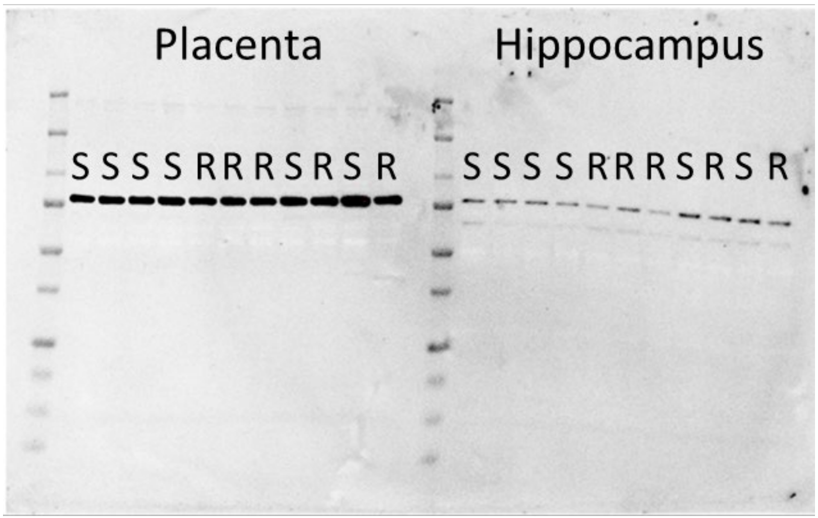

Figure S2: Full Western blot (inverted greyscale image) for ASIC2a staining. On the left, placental samples were loaded. On the right, hippocampal samples are shown. The ASIC2a bands were observed at ~76kDa.

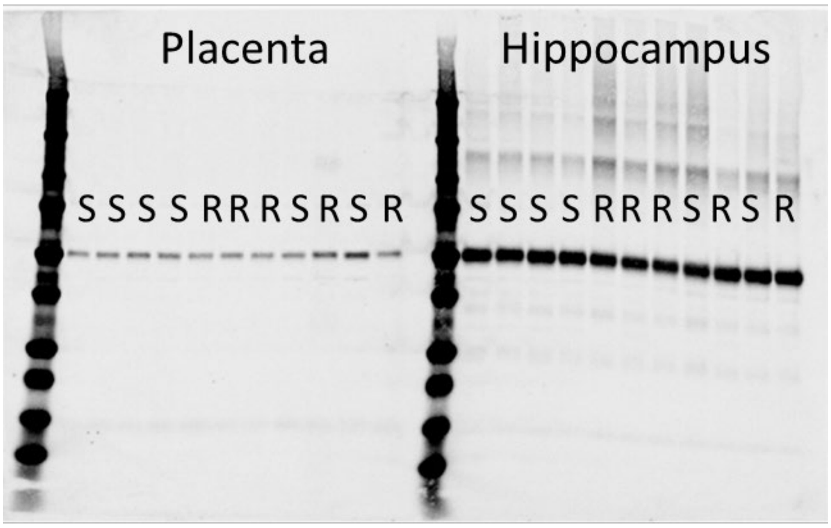

Figure S3: Full Western blot (inverted greyscale image) for alpha tubulin staining. On the left, placental samples were loaded. On the right, hippocampal samples are shown. The antibody recognized a band at 50kDa. Extra bands were visible in the hippocampal samples.

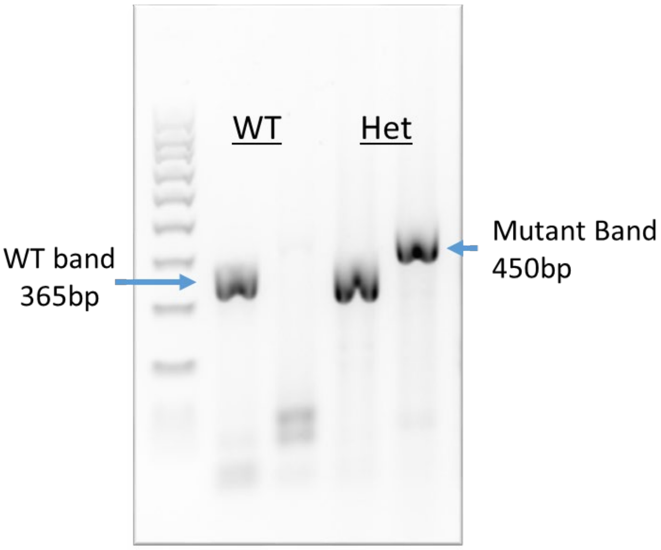

Figure S4: Representative bands from ASIC2a genotyping. ASIC2a+/+ mice have only the WT band (365bp) while ASIC2a+/- mice have the WT band plus the mutant band (450bp).
